# Recent Advances in Targeting of Breast Cancer Stem Cells Based on Biological Concepts and Drug Delivery System Modification

**DOI:** 10.34172/apb.2020.042

**Published:** 2020-05-11

**Authors:** Zeynab Aaliyari-Serej, Ayyub Ebrahimi, Balal Barazvan, Abbas Ebrahimi-Kalan, Khalil Hajiasgharzadeh, Tohid Kazemi, Behzad Baradaran

**Affiliations:** ^1^Department of Applied Cell Sciences, School of Advanced Medical Sciences, Tabriz University of Medical Sciences, Tabriz, Iran.; ^2^Immunology Research Center, Tabriz University of Medical Sciences, Tabriz, Iran.; ^3^Student Research Committee, Tabriz University of Medical Sciences, Tabriz, Iran.; ^4^Department of Molecular Biology and Genetics, Faculty of Arts and Sciences, Halic Uuniversity, Istanbul, Turkey.; ^5^Department of Basic Sciences, School of Medicine, Gonabad University of Medical Sciences, Gonabad, Iran.; ^6^Department of Neurosciences and Cognition, School of Advanced Medical Sciences, Tabriz University of Medical Sciences, Tabriz, Iran.; ^7^Department of Immunology, Faculty of Medicine, Tabriz University of Medical Sciences, Tabriz, Iran.

**Keywords:** Breast cancer, Cancer stem cells, Cell signaling, Multi-drug resistance, Nanomedicine

## Abstract

Breast cancer with various biological diversity known as the common reason of death in the world and despite progress in novel therapeutic approaches, it faced with failure and recurrence in general. Recent clinical and preclinical statistics support cancer stem cells (CSCs) hypothesis and its similarities with normal stem cells. Evaluation of related paper conclude in significance finding in the further characterization of CSCs biology such as surface biomarkers, microenvironment regulatory molecules, cell signaling pathways, cell to cell transition and drug efflux pumps to overcome multidrug resistance and effective therapy. Emerging novel data indicate biological concepts in the base of unsuccessful treatment. A powerful understanding of the cell signaling pathways in cancer and CSCs topics can be led us to define and control treatment problems in cancer. More recently nano medicine based on drug delivery system modification and new implications on combinatorial therapy have been used to treat breast cancer effectively. The aim of this review is focus on CSCs as a potential target of cancer therapy, to overcome the limitation and problems of current therapeutic strategies in cancer.

## Introduction

### Breast cancer biology

**Figure 1:**
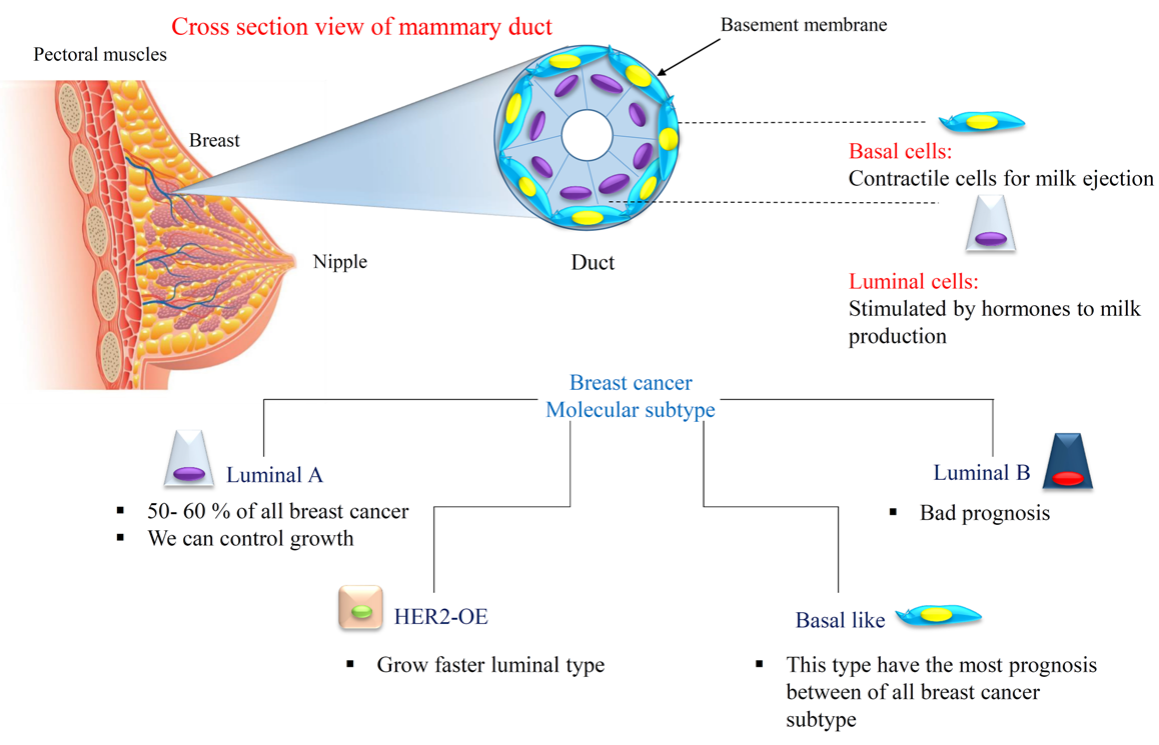
Mammary gland and the cellular variety in breast cancer.

Breast cancer is the most common malignancy and the 5th cause of cancer related death among the US women.^1,2+^ Breast cancer is a disease with histological, molecular and epidemiological heterogeneity which this heterogeneity creates big challenges to the development of effective cancer treatment.^3^ There is a lot of biological diversity in the breast cancers that occur because of variations in transcriptional programs. To distinguish patients with a high risk of progression, breast cancers are classified into subtypes according to gene expression profiles:

1) Luminal A
2) Luminal B,
3) Human growth factor receptor 2 overexpressing (HER2-OE), and
4) Basal-like tumors as shown in Figure 1.^4-6^

In the breast cancer treatment, the recurrence and failure cause a big obstacle in treatment of the cancer. To reduce the cancer-related mortality rate, studying the biological properties of cancer and discovering new therapeutic approaches can be helpful. Several mechanisms cause to chemoresistance like alterations in apoptotic signaling pathways, metabolic enzymes defection, mutations in tumor suppressor genes, increased drug efflux pumps, reduced drug uptake and tumor microenvironmental changes in response to therapy.^1,7^ Despite progress in common treatment strategies of cancer like chemotherapy, radiotherapy, and surgery, an untreatable population of tumors remains that metastasize to distant organs. These population displays stem cell properties that we focused more in next part.^8^

## Normal and cancer stem cells in breast

**Figure 2:**
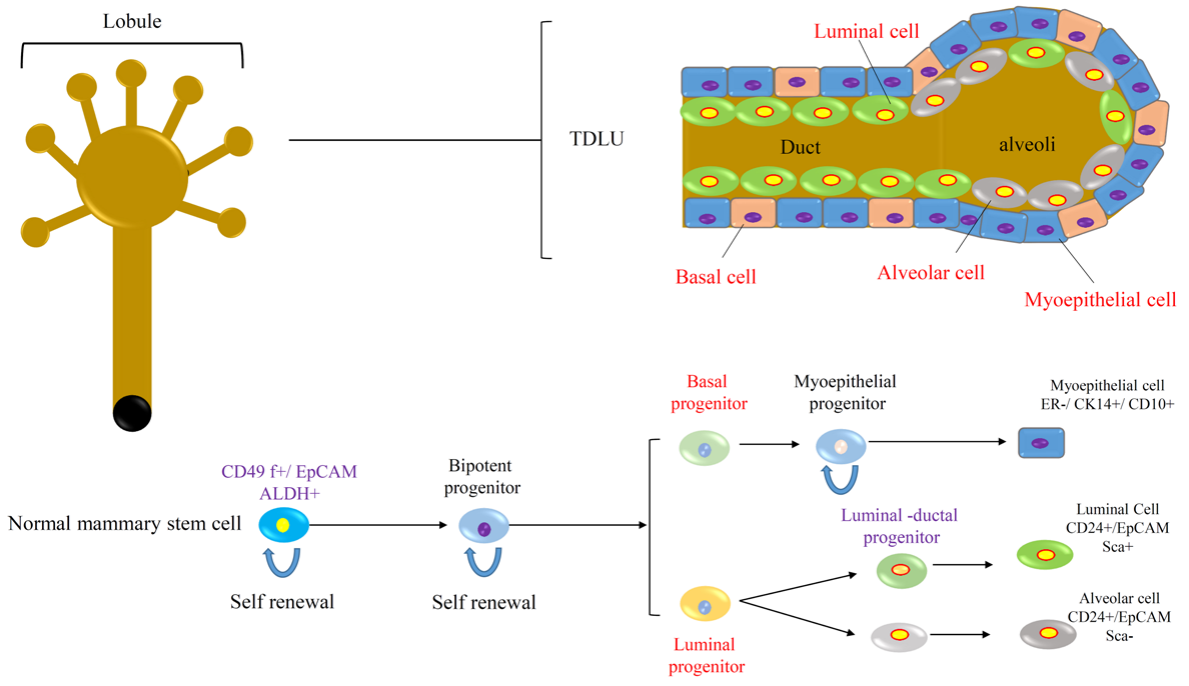
Normal mammary gland stem cells produced various types of cells and differentiation processes.

The presence of breast stem cells has been hypothesized from the evidence that the breast tissue can be regenerated after transplantation of epithelial tissue in mice. The epithelial and mesenchymal cells composed the breast tissue and formed terminal ductal-lobular units (TDLU).^9^ Stem cells in the normal breast tissue produce early and late progenitors, that these progenitors finally differentiate into (Ι) the luminal or alveolar epithelial cells; (II) the ductal epithelial cells and (III) the myoepithelial cells (Figure 2).^8,10,11^

The normal breast stem cells are CD49f +/EpCAM- that are capable of self-renewal and differentiation to various types of breast tissue cells. According to recent studies, the breast stem cells can be enriched within a CD49f +/EpCAM- population with a basal cell specification.^12^ The aldehyde dehydrogenase (ALDH) enzyme is another normal breast stem cell marker that plays a functional role in stem cell differentiation. Studies of the normal breast tissue show that about 6% of the epithelial cells in the TDLU were ALDH+ and these cells can generate mammospheres in the suspended culture condition.^13^ Another subset of normal breast stem cell was found that are keratin K19 negative and the part of these cells increased under proliferative conditions such as epithelial hyperplasia; so, they can be the origin of breast cancers.^14^

Cancer stem cells (CSCs) firstly were discovered in acute myeloid leukemia and they have become an important part of research as a potential target for cancer therapy. The origin of breast CSCs is the mammary multipotent stem cells with genetic defects that affect pathways related to self-renewal and differentiation.^15^ So, the origin of these cells is important for the prevention, early detection, and breast cancer therapy.^16^ CSCs have the similarities with normal stem cells like being quiescent, multipotency and self-renewal capacity that these specifications helps preserve the tumor.^17^ CD44+/CD24low/- and ALDH are common CSC markers that are the same with normal stem cells. Common markers of BCSCs have been briefed in Table 1. In addition, embryonic stem cell markers and transcriptional factors expressed by CSCs. They are including of stellar, rex-1, nestin, and H19, β-catenin, OCT4, NANOG, and SOX2.^18,19^ During carcinogenesis, these factors reprogram differentiated tumor cells into undifferentiated stem-like cells.^14^ Breast cancer CD44+ cells are like to normal stem cells and they show basal-like cells properties. But CD24+ cells have shown similarity with luminal cells. In the breast cancer cell lines, we can find these two cell types which demonstrating a similar hierarchy to tumor cells.^20^ BCSCs can survive in the therapeutic condition in comparison with the more differentiated tumor cells.^21^

**Table 1:** BCSC common markers

**Marker**	**Cell Line**
CD44+CD24-/low	MCF7, MDA-MB231,361, and 468, SKBR3, BT-549 and HCC1937
CD44+CD24-/low ALDH1+	MDA-MB231, 453, and 468, ZR-75, SUM149 and 159, SKBR3, and HCC1954
ABCG2+ CD44+CD24-/low	MCF7 and MDA-MB231
EpCAM+ CD44+CD24−/low	MDA-MB231, MCF7, SUM149 and 159
CD133	MDA-MB231, MCF7, ZR-75
ABCG2	HCC1937
CD29	MCF-7
CXCR4	MCF-7, Mouse 4T1, 4T07, 168Farn, and 67NR

## Cancer stem cell/stem cell regulatory/oncogenic molecules

**Figure 3:**
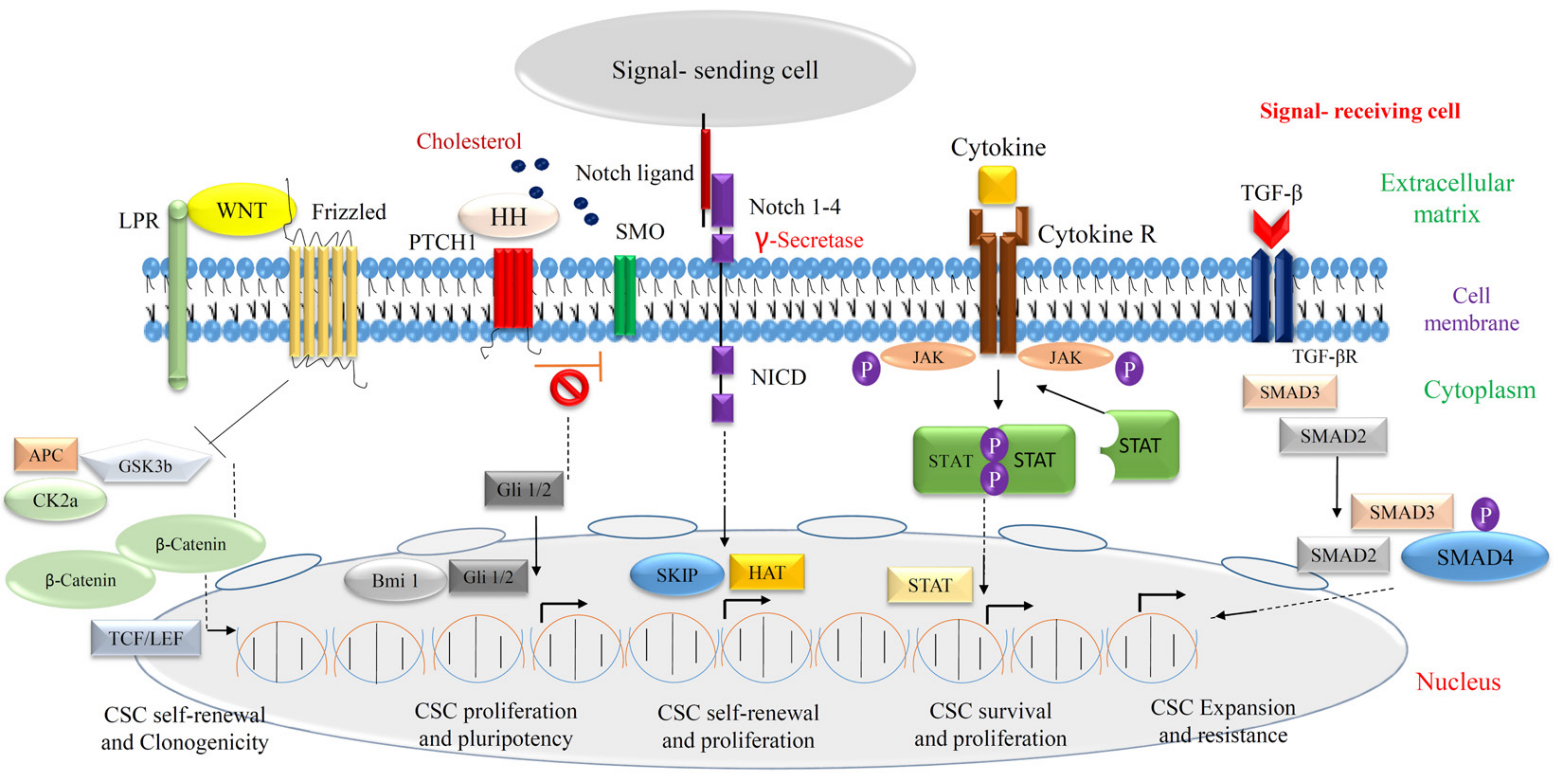
Cell signaling in breast cancer/CSCs.

Breast cancer-related deaths in the United States make a dramatic number (40 000) every year, and involved with the metastatic condition generally.^22^ CSCs may play a central role in contributing to this circumstance and understanding critical molecular components of cell signaling pathways is the priority of defining new therapeutic strategies. Here some important molecules participated in the regulation of network signaling concept that have been shown in Figure 3 like: Wnt/b-catenin, Notch, Hedgehog (HH), epidermal growth factor (EGF)-like and it receptors (EGFR)/Neu, transforming growth factor beta (TGF-β), leukemia inhibitory factor as a the Jak/Stat signaling activator, integrins, SDF-1/CXCR4, telomerase, the insulin-like growth factor-1 system, Prolactin/growth hormone (GH) and ER/PR (Table 2).^23^ Numerous studies consider the roles of mentioned molecules and their signal transduction, but it seems there is a much more complicated context to reach an exact treatment without gross side effects.

**Table 2:** Signaling pathways in CSCs

**Signaling pathway**	**Roles in CSC**
Wnt	It involves in breast tissue selfrenewal, clonogenicity and tumorigenesis, mediate radio-resistance
Notch	It involves CSC self­renewal, breast cancer initiation and progression, radiation resistance in breast cancer cell lines
Hedgehog	It involves in control the EMT, metastasis, pluripotency and proliferation of breast CSCs
TGF-β	It involves in expansion of CSCs, resistance of CSCs
Jak/Stat	It regulates the survival and proliferation of CSCs

### Wnt/b-catenin signaling pathway in breast cancer

One of the key regulatory and embryonic originated molecules in the cancer formation and progression is Wnt and its ectopic expression which increased the breast stem cells that occurred by alteration of the epithelial hierarchy. Besides of embryological approach for Wnt function, it is involved in the function of some somatic cells such as the brain, blood, lung and mammary gland. The abnormal function of Wnt can lead the cell to tumor formation and expands breast stem cells.^4,24^ Previous studies showed Wnt also involved in chemo- and radio-resistance of breast CSCs.^4,11^ The subpopulation of CSCs is maintained by the employment of Wnt ligands by periostin, a component of the fibroblast’s extracellular matrix. Wnt signaling in tumor cells induces its expression and colonization in the context of lung tissue. Despite inhibition of the Wnt function show poor progression and metastasis in some cancer, but we need more information on the Wnt signaling pathway to finalize the therapeutic approach.^25^

### Notch signaling pathway in breast cancer

Like Wnt, deregulation of Notch as a very important embryonic molecule involved in tumor progression and self-renewal of CSCs in adult which conclude in mammosphere formation.^26^ Inhibition of Notch receptors signaling pathways affected mentioned items as a novel therapeutic approach. Generally, Notch plays so much role in the developmental biology of breast tissue.^27-29^ Various subtypes of Notch such as Notch1 and Notch4 have a different function in cleaved/activated form. For example, upregulation of the Notch 4 induced metastatic conditions in the breast epithelial tissue.^30^ The differential expression of Notch4 versus Notch1 receptors in breast CSCs can suggest different characters for each receptor and so our therapeutic strategies.

### HH signaling pathway in breast cancer

The mentioned molecules have principle roles in the embryonic period, but more important functions can be found for some of them in abnormal and pathologic conditions. HH pathway implicated in the viability of adult tissues. Regards knowledge about the role of deregulation of HH in cancer progression, there are very few studies links this role in CSCs too.^31^ But more recently, it has been shown not only downstream effectors of HH signaling such as polycomb group involved as a central regular in self-renewal of adult stem cells but also participated in CSCs pluripotency.^32,33^ Epithelial-mesenchymal transition (EMT) procedure as a key factor in tumor progression has been affected by the HH signaling pathway and could be defined as a therapeutic approach in pancreatic cancer.^34^ Until now we numbered characteristics of some of the most important molecules involved in developmental biology and abnormal issue in the body. But in continue, we showed another essential molecule that affected the growth of tumor cells passively or negatively.

### TGF-­β signaling pathway in breast cancer

TGF-β is a multifunctional cytokine, produced by white blood cells and affect stemness properties through the expansion of CSCs in breast cancer. More recently a study declared that chemotherapy decline the growth of tumoral cells in breast cancer probably via CSCs expansion modification through the alteration of IL8 and TGF-­β signaling pathways.^35^ TGF-β signaling synchronization with the WNT pathway in breast cancer, induce EMT and cause more progression of the tumor. Several molecules have been involved in cancer metastasis but the role of TGF-β is essential because of the interaction of other immune cells. Indeed, the dysregulation TGF-β detected in several types of cancers especially having a role in progression to malignancy of breast cancer. But it seems a careful clinical study needs to approve this function completely.^36^

### Jak/Stat signaling pathway in breast cancer

Some of the proposed molecules having a different role in animals and humans. The Jak/Stat in Mouse embryonic stem cells promote self­renewal and pluripotency but in human leukemia inhibitory factor have not this function. One of the known activated molecules of this pathway, named STAT3 promotes tumorigenesis and increases the survival/proliferation of breast CSCs.^25^ It was possible to assume that activation of JAK/STAT signaling pathway is associated directly with tumor fate in term of therapeutic approach.

### Epithelial-mesenchymal transition induced formation of CSCs

**Figure 4:**
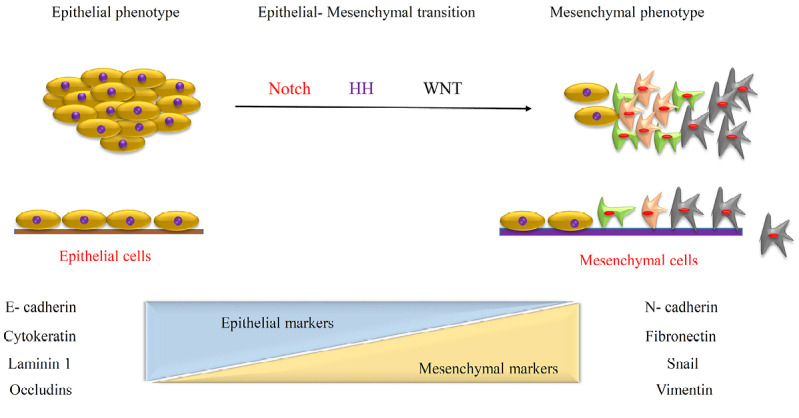
EMT induced formation of CSCs through the various molecular expression.

EMT cause loss of cellular polarity and cell-cell interaction and adhesion and gain stem cell properties similar to mesenchymal cells, with migratory and invasive characteristic.^37,38^ The similarity between CSCs and stem cells shows a lot of cue to clinic and experiment aims. EMT normally occurred during the development of a fetus and indeed it is one of the most important events, which has been detected in CSCs growth and tumorigenic process.^39^ Based on embryological understanding facts and similarity we can use those molecular approaches and signaling transduction in defining of novel therapeutic strategy. There are several known cells signaling such as Wnt, Hedgehog and Notch which involved in both CSCs and normal stem cell viability and renewal. In contrast expression of genes related to inducing epithelial differentiation, can target as a good alternative in treatment (Figure 4). For example, forced expression of key genes like E-cadherins leads to a high level of CD44 and low level of CD24. CD8+ T-cells change the expression pattern to epithelial genes. TGF-β involved in suppression of tumor progression through induction of apoptosis by expression of special molecules which leads to CD44 and CD24.^40,41^

## Novel therapeutic strategies targeting CSCs

**Figure 5:**
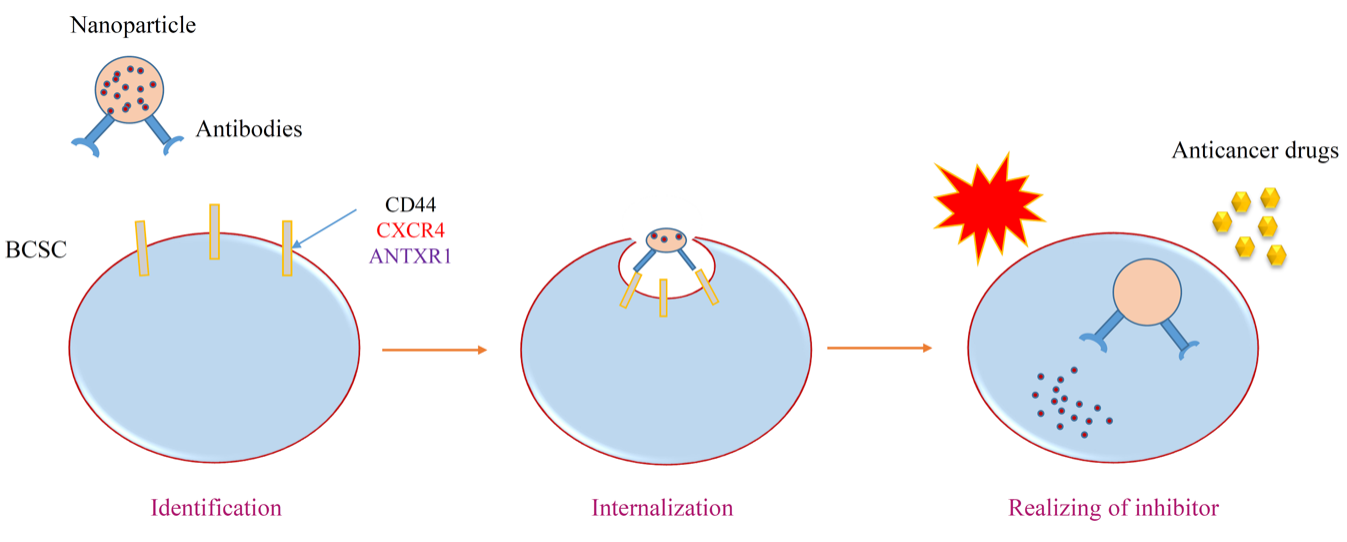
Nanomedicine and its role on sensitizing of tumor cells resistance to chemotherapy.

Recently CSC hypothesis is defined as a pivotal axis of therapy in the clinic. It is completely cost-benefit, the researchers focus on CSCs instead of cancer, because of the minority of CSCs as a main source of tumor growth in comparison with cancerous cells with an awareness of complexity in reaching to CSCs.^42^ The problem of multi-drugs resistance (MDR) in cancer treatment produced therapeutic limitations based on CSCs renewal and progression after the onset of the therapeutic procedures. Thus, focus on CSCs is so critical to improving the quality of treatments in the clinic. In this review, several strategies have been designed to gain final goal in CSCs biology such as developmental anatomy principles, EMT, surface biomarkers recognition/targeting, oncogenes profile association, cell signaling in CSCs, the role of pumps in drugs efflux and resistance, and microenvironment and immune cell behavioral. Apart from these items, nanotechnology and its recent powerful advance also use in combinatorial therapy (Figure 5).^17^

## Targeting surface biomarkers of CSCs in breast cancer

Of the clinical and basic sciences significances and defining more specific/affective treatment is the recognizing of the surface biomarkers of CSCs compared to normal stem cells. Exact information about biomarkers means the intervention of certain signaling transduction, which makes CSCs good candidates in cancer treatment.^43^

### CD44, CD133 and EpCAM as key biomarkers in therapeutic strategy

Beside of CD44 and CD133, there are some other CSCs common surface biomarker (see Table 1). But CD44 as a membranous CSCs biomarker located at the top of the list for mentioned interventions and communication with the extracellular and microenvironment ligands. Previous studies showed.^43^ Another biomarker in tumoral cells is EpCAM. In most of the EpCAM+/CD44+/CD24- cell populations, humanized antibodies based on mentioned biomarkers antibodies have been shown successful results in experiment and clinic. More recently, amplification of chemotherapy has been detected in combinatorial therapy of salinomycin and paclitaxel load on HA-decorated nanoparticles, to block CD44+ in CSCs.^44,45^ More recently, amplification of chemotherapy has been detected in combinatorial therapy of salinomycin and paclitaxel load on HA-decorated nanoparticles, to block CD44+ in CSCs.^46^

In xenograft model MDA-MB-231, anti-CD133 mAbs loaded with polymeric nanoparticles also provide chemotherapeutic drugs good internalization. CD133 is trans-membranous protein Known also as prominin-1 which express in the vast majority of stem cells and if be positive definitely simulates CSCs properties and it is approved in an animal model of cancer in NOD/SCID mice. Here also CD133-target nanoparticles which loaded by anti-tumoral drugs was established. This complex leads to tumor cells location by anti-CD133 antibody.^47^ Indeed, combinatorial therapy against intrinsic resistance might be a more successful method in cancer treatment and showed decreasing tumor size and CSCs populations.^43^ Beside utilizing of nanomedicine as a novel therapeutic method, more recently development of CD133-specific RNA aptamers to the identification of the CD133 and AC133 epitope with high sensitivity in various types of cancer cell lines defined as a good alternative in tumor internalization and retention in comparison with using antibodies in 3D tumor sphere model.^48^ However, CD133 as an early biomarker can be critically defined in human stem cells evaluation. Thus, novel therapeutic strategies focus on CD133 to prevent CSCs growth in tumor with awareness of probable induction of unpredicted myelosuppression. But it seems more research is needed to criticize the mentioned target as a clinical application in practice and theory. Conclusion of certain role of CD133 in CSCs eradication must be supported with valid consideration of CD133+ cancerous cell behavior pattern.^49,50^

### ALDH1: prognostic significance in CSCs control and breast cancer treatment

Modification in the various enzymes of the CSCs like ALDH1 function which mediated the oxidation of aldehydes to carboxylic acid in the cell can be useful in the sensitizing of the tumor cell. Diethylaminobenzaldehyde (DEAB)) as a specific direct ALDH inhibitor and ATRA (The differentiation agent all-trans retinoic acid (ATRA)) indirectly target the activity of ALDH by the retinoic acid (RA) pathway.^14,51^ Critical issue in biomarkers topic is the similarity of some surface markers of CSCs with normal stem cells and must be aware of its side effects through the recognition of signaling pathway in detail. Decreasing in tumor size and inhibition of its growth never show the CSCs percentage diminishing. It seems novel therapeutic strategies must be focused on this issue.^43^

## Targeting cell signaling in CSCs that regulate self-renewal and differentiation

**Figure 6:**
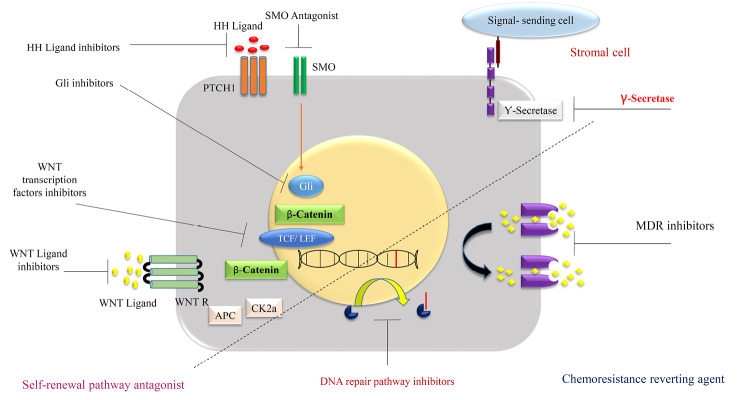
Different strategies to target the BCSCs.

Signaling pathways and deregulation of its modalities such as Stat3/mTor, Hedgehog, Notch and Wnt/-catenin cascade defined as a new therapeutic strategy in the prevention of cancer extension, especially CSCs growth behaviors modification (Figure 6). Multiple aspects of cell signaling make this alternative so susceptible and targeting that kind of treatment item seems so difficult to utilize in the clinic. A lot of new generation drugs withdraw after several experimental and clinical trials, in terms of the complexity of signaling pathways.^17,43^ Mentioned signaling items directly involved in the developmental of the body during intra uterine life and similarity of normal stem cells to CSCs reveal more knowledge in both of them mutually.^52^ The notch signaling pathway and its cascade in multiple biological aspects finally affected CSCs stemness, MDR, and hypoxia inducible factor (HIF) pathway.^53^ On the intermediated modalities in this cascade such as γ-secretase inhibitors and antibodies directed showed accepted resulted in a clinical trial in various types of cancers.^54^ It is critical to know about the diversity of Notch signaling to manipulee of the final events in special cancer like breast cancers. Utilizing of Notch signaling inhibitors in oncology related to many alternatives such as type of cancer, exact pharmacodynamic effects identification and mechanistic understanding of it signaling. For example, CD44 as a biomarker in CSCs is Wnt signaling target and knockdown of CD44 diminished tumor formation.^55^ In another study administration of an antagonist of Wnt decreased mammosphere formation.^56^

## Microenvironmental modalities control CSCs fate

**Figure 7:**
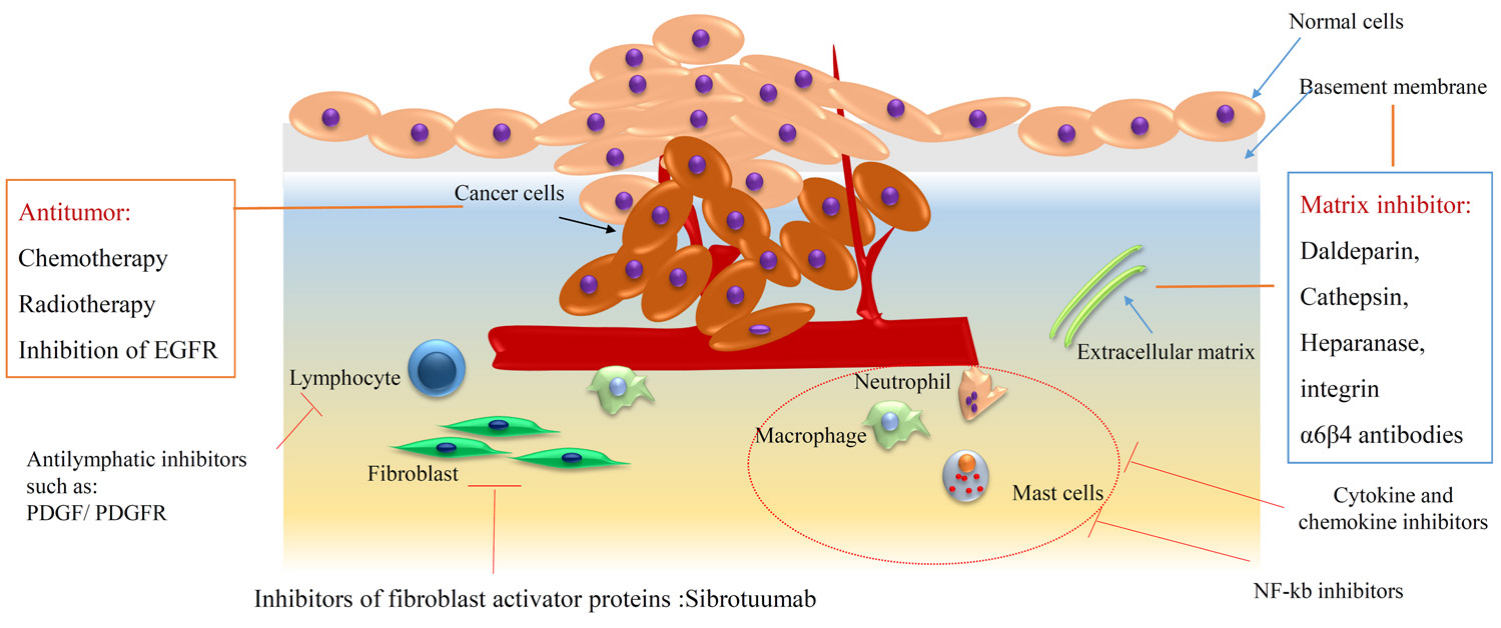
The role of immune cells interaction in breast in cancer progression.

Preservation of CSCs in the tumor, depends on the microenvironment of cancer and recently it is defined as a novel pharmaceutical target. Indeed, a suitable niche has been provided by (a) immune cell secretion, conclude in chronic inflammation, (b) perivascular space involved in proliferation and differentiation and, (c) finally routine hypoxia which is known as a critical issue in the growth of CSCs (Figure 7). The Stat3/ NF-κB signaling pathway is activated by secreted cytokines such as IL-1β, IL-6 and IL-8 and modification in a positive way leads to CSCs stemness preservation, neovascularization and metastasis.^57,58^ Molecular modification of the tumor microenvironment can block the growth of CSCs. Repertaxin as an inhibitor of IL-8 increase chemotherapy efficiency and diminishing tumor size.^59^ Here also combinatorial therapy makes the curing process more effective than before multiple biological approaches by using IL-6 and IL-8 inhibitors or HIF pathway blocking.^60^ It seems there is more detail information about signaling pathways needed to overcome CSCs microenvironment suitability.

## Apoptosis and differentiation pathways in CSCs targeting therapeutic approach

One of the most critical issues during primary stages of cancer, development and metastasis is the impairment of apoptosis procedures. Indeed, the cells in the normal tissues of the body without the apoptosis process be cancerous definitely. Thus, internally or externally induction of this onset of the apoptotic signaling pathway can be defined as a reliable method in novel cancer therapeutic studies. Modification in apoptotic genes and receptors by synthetic or natural materials have been used in several studies. P53, bcl-2, ras and c-myc cell cycle regulating genes and death related receptors like CD95, the tumor necrosis factor (TNF) receptor, trimeric human tumor necrosis factor-related apoptosis-inducing ligand (TRAIL) are the part of apoptotic induction targets which finally activated caspase 8 and then caspase 3 which finally conclude in permeabilization of mitochondrial membrane and cell death.^61^ Combinatorial therapy with cell death agents like engineering in mesenchymal stem cells to express TRAIL based viral vectors^62^ and anticancer drugs like cisplatin, daunorubicin and cytarabine, inhibition on transcription factor of NF-kB by proteasome inhibitor is critical in tumoral cells survival.^63^ Suppress growth of CSCs, trigger and induce apoptosis through inhibition of the Wnt signaling pathway in various types of cancers.^64^

Modification in apoptosis mechanism beside anti-cancer drugs provide a good preclinical study goal, but there is some issue in specific effects, side effects problems and toxicity of the mentioned approach most be solved to allow utilization in the clinic.

## Efflux-mediated drug pumps in MDR of cancer therapy

MDR in cancer treatment, especially chemoresistance highly related to overexpression of ABC transporter as drug efflux pumps (P-glycoprotein (P-gp)). Thus, inhibition of the function of transporter simply can be recognized as an anti-MDR procedure. But low specificity and high side effect defined as an obstacle in this alternative. Although some of these inhibitors like Vardenfl showed the accepted result in P-gp blocking and increasing anti-cancer drugs like (paclitaxel and vincristine) intracellular concentration.^65^ Another alternative to the silence of P-gp is the use of RNAi such as siRNA^66^ alone or in the combination with nanoparticles application can simulate the effects of inhibitors of P-gp. For example, utilizing conjugated copolymers with ritonavir (ABC transporter inhibitor) increase the sensitivity and mortality of drug-resistant tumoral cells through enhancement in the cytotoxicity of doxorubicin in leukemia. Combinatorial therapy by application of nanotechnology in this field provides promise to overcome CSCs resistance to routine drug and classic treatments.^67^

## Approaches to manipulation of miRNA expression in breast cancer

To good monitoring and diminishing the risk of cancer progression, scientists attempt to define a new powerful method in the cellular and molecular levels. Early detection by sensitive and specific biomarkers placed on top of the list. Body fluids like peripheral blood contain molecular targets called nucleic acids such as DNA, RNA and miRNA. Thus genetic, epigenetic modification and microenvironmental alteration as a very novel approach has been recognized in this field.^68^ Among them, circulating miRNA as a valuable molecular tool with its stability and approximately noninvasive isolation procedure, make it a valuable alternative in diagnosis, prognosis and even a good target for monitoring of anti-tumor interventions response.^68,69^ During the onset and progress of cancer, various types of miRNA can be upregulated or downregulated in different stages of the diseases. Deliberate dysregulation of mentioned oncogenic miRNA with mimic miRNA leads to alteration in tumor-suppressive genes and trigged or blocking a signal transduction pathway conclude in defining a new therapeutic strategy with knowledge about the complication of this procedure. Previous studies with sequencing and PCR techniques showed some miRNAs expression are modified in a lot of the diseases including breast cancer and profiling of miRNA has been considered to determination and evaluation of disease progress.^70^ MiRNA based treatment goes to establish a feasible approach to enhance current anti-cancer therapeutic strategies. But there are several obstacles to reach the final aim before clinical utilization such recognition of differential expression pattern of miRNA that involved in particular cancer on experimental animal model, degradation and instability problems solving in in-vivo, target base function to avoid side effects on normal tissue.^68^ For example, Yu et al. showed that in SKBR3 cells, expression of some miRNA like let-7, miRNA-16, 107, 128 significantly changed compared with differentiated cells in breast tissue context.^71^

## Breast CSCs treatment approach by various differentiation inductive agents

In general, the target of cancer therapy is the differentiated cells but recently induction of CSCs differentiation is established as a novel method to decreasing tumoral cells population. These approach handle by some differentiative inductive agents like: histone deacetylase inhibitors (in several hematologic malignancies,^72^ retinoic acid (its analogs ATAR use in acute promyelocytic leukemia treatment,^73,74^ tyrosine-kinase, miRNAs (miR-100) and even signaling pathways modification (Akt/mTOR and P53).^75^ Knockdown of special genes or proteins such as pancreatic differentiation 2 (PD2) protein in CSCs affected cell viability, trigged apoptosis process^76^ and decrease expression of stem cell markers. Combinatorial therapy with a focus on differentiated cells and CSCs by various alternatives can be so useful to define a new therapeutic strategy in the cancer treatment field.

## The role of inflammatory cells in breast CSCs

The correlation between inflammation and neoplasia has been considered by several studies, which showed that chronic inflammation of pancreatic to helicobacter pylori infection associated with gastric, colorectal, bladder, pancreatic, liver, lung and even cervical cancers^77,78^ while lymphocyte infiltration particularly T helper and T cytotoxic showed good prognosis in tumors, in chronic inflammation are the innate immune cells which accompanied poor prognosis. Indeed, the cytokines of mentioned cells produce a good niche for metastasis of cancerous cells.^78^ These immune cells also altered tumor cells genetically by the production of free radicals, which form peroxynitrate (DNA damage agent).^79^ The gist of these studies can highlight the fact that that lower chronic inflammation equals a lower risk of cancers. Epidemiological research on nonsteroidal anti-inflammatory drugs (NSAIDs) administration provides more good results in cancer treatment,^80^ albeit side effects of NSAIDs not be ignored on the cardiovascular system and so need to develop of a new therapeutic approach. Apart from inflammation factors, proinflammatory factors, TNF-α,^81^ IκB kinase, the upstream NF-κB activator kinase, interleukins 1, 6, 8 and certain chemokines and their receptors inhibitors^82^ can be defined as potential targets for cancer therapy. Another strategic alternative is protumorigenic factors to recognize and modify them to reach the final goal of treatment in cancers.^83^ The future direction seems must be the focus on immune cell’s genetic or epigenetic modification to gain an accepted therapeutic approach is the change of tumor-infiltrating immune cells gene expression through the cytokine cells balance alternation. Immune tolerance can be changed by cytokines effectors administration to reduce immune system response enhancement, but there is a very important part and it is side-effects of utilizing these methods. Therefor final strategy must be localized to decrease the toxicity of the mentioned therapeutic alternatives. Recently studies focus on the modulation of immune cells functions by CSCs microenvironment. Chronic inflammatory condition is a key factor in the tumor microenvironment that leads to proliferation and metastasis of the cancerous cells through the immunosuppressing of immune cells like T cytotoxic and Natural killer cells.^84-86^ In some cases, dendritic cells (DCs) anti-tumor antigens are masked from tumor cells by tolerizing antigens presenting cells. Indeed, DCs stimulate T cytotoxic.^87^ Immune cells (tumor-associated macrophages (TAMs), tumor-associated neutrophils (TANs), and myeloid-derived suppressor cells (MDSCs)) recruited by tumor cells through the cytokines and chemokine secretion. TAMs secrete TGF-β with a role in immunomodulation and immunosuppression, recruits T regulatory cells (Tregs).^88^ MDSCs are bone marrow derived origin, also involved in cytokines secretion like (IL6, TGF-β) and also recruit proinflammatory secreted cells such as T helper 17 cells which improve immunosuppression.^86^ TANs and TAMs cause cancer progression with the breakdown of ECM lead to metastasis and tumor angiogenesis.^84,86^ These cell’s exosomes can be internalized mRNAs and microRNAs into different cell types^89^ for tumor cell proliferation, metastasis and chemoresistance through the drug efflux pumps transporter.^90^ In the inflammatory TME, secretion of TNF-α by TAMs and CD4+ T cells, involved in upregulates of NF-kB signaling pathways. Downstream cell signaling continuously caused in self-renewal and finally promote migration and invasion of CSCs. The conversion of non-CSCs to CSCs may be induced by stemness and EMT correlation.^91^

## Breast CSCs and immune modulation of microenvironment

Besides of very clear role of CSCs in proliferation, metastasis and therapeutic resistance of tumor cells, more recently the role of CSCs in extent of the tumor through diminishing immune cells surveillance intra tumor microenvironment has been investigated. Additionally, CSCs are potent to escape from immune cells by alteration of immunogenicity. Furthermore, an experimental model showed CSCs directly related to the progression of tumor cells and can be useful if we destroyed or ablated those.^92^

CSCs delayed or cut the response of immune cells by secretion of immunosuppressive factors, mimicking of antigen-presenting cells (expression of MHC I) and the co stimulator molecule (programmed death-ligand 1, PD-L1) inhibition or lack of co-stimulatory molecule ( CD40, CD80, and CD86) activation which lead T cells to abnormal immune response to a specific antigen. The secretion of galectin-3 by CSCs triggered an apoptotic process in T cell.^93^ A very complicated response and organized gene expression and secretion of particular effectors (TGF-β) of CSCs conclude in attenuating MHCII and depleting intratumor immune cells finally even through the expansion of T regulatory cells.^94-96^ In contrast, not only tumor supportive phenotypes cells (TAMs) recruited through periostin secretion to the microenvironment of the tumor but also macrophage migratory factors (colony-stimulating factor-1 (CSF-1), macrophage inhibitory cytokine 1 (MIC-1) and C-C motif ligand 2 (CCL2) induced by CSCs make the condition worse than before. These factors lead to a monocyte context to the immune suppressive M2 phenotype.^97^ As mentioned above macrophage is critical for maintains stemness in CSCs in breast via interactions of CD90 (Thy1) and EphA4 and induction of downstream signaling such as Src and NF-κB. Indeed NF-κB is so important for the recruitment of macrophage in breast cancers. The self- renewal of CSCs in breast tumor increased by IL6 that activated signaling pathway of STAT3. It seems more studies are required about the reciprocal interaction of CSCs and immune cells in micromovement of the breast cancer tumor and its progression behaviors.^98^ insert clinical point, mentioned future studies suggestion and point your limitation of your study.

## Conclusion

Breast cancer is a disease with histopathological and epidemiological heterogeneity. Here we discussed about various alternatives and biological characteristic concept of CSCs include targeting surface biomarkers, targeting cell signaling, microenvironmental modalities alteration, apoptosis and differentiation pathways, efflux-mediated drug pumps in MDR, manipulation of miRNA expression, differentiation inductive agents, inflammation, immune modulation of microenvironment, etc in CSCs and breast cancer treatment. For example, exact information about biomarkers means the intervention of certain signaling transduction, which makes CSCs good candidates in cancer treatment or it seems more detail information is need about signaling pathways to overcome CSCs microenvironment suitability. Modification in apoptosis mechanism beside anti-cancer drugs provide a good preclinical study goal, but there is some issue in specific effects, side effects problems and toxicity of the mentioned approach most be solved to allow utilization in the clinic.Numerous experiments and clinical studies consider the roles of mentioned pattern, but it seems there is a much more complicated context to reach an exact treatment without gross side effects. It seems so much careful clinical study needs to approve this function completely. Indeed, knowledge about stem cells and particularly CSCs can be led us to define a novel therapeutic strategy based on new technology (nano biomedicine) and CSCs interventions. In fact, nanotechnology and its recent powerful advance also use in combinatorial therapy. Finally, emerging novel data indicate biological concepts in the base of unsuccessful treatment. A powerful understanding of the cell signaling pathways in cancer and CSCs topics and their interaction with other biological modalities can be led us to define and control treatment problems such as metastasis and chemoresistance. It will be critical for the developing of effective anti-tumor drugs, utilizing nanomedicine.

## Ethical Issue

Not applicable.

## Conflict of Interest

The authors declare that they have no relevant affiliations or financial involvement with any organization.
